# Eribulin-Induced Peripheral Neuropathy in Locally Advanced or Metastatic Breast Cancer: Final Analysis of the Prospective Cohort IRENE Study

**DOI:** 10.3390/cancers17030457

**Published:** 2025-01-28

**Authors:** Marcus Schmidt, Tobias Hesse, Oliver Hoffmann, Bernhard J. Heinrich, Tjoung-Won Park-Simon, Eva-Maria Grischke, Rudolf Weide, Harald Müller Huesmann, Kerstin Lüdtke-Heckenkamp, Dorothea Fischer, Cosima Zemlin, Matthias Kögel, Yan Jia, Helga Schmitz, Christian Engelbrecht, Christian Jackisch

**Affiliations:** 1 Department of Obstetrics and Gynecology, University Medical Center Mainz, 55131 Mainz, Germany; 2 Department of Obstetrics and Gynecology, Sana Klinikum Offenbach GmbH, 63069 Offenbach, Germany; 3 Department of Gynecology, Agaplesion Diakonieklinikum Rotenburg gGmbH, 27356 Rotenburg (Wuemme), Germany; hesse@diako-online.de; 4 Department of Gynecology and Obstetrics, Universitätsklinikum, 45147 Essen, Germany; oliver.hoffmann@uk-essen.de; 5 Haematologie und Onkologie im Zentrum MVZ GmbH, 86150 Augsburg, Germany; bernhard.heinrich@hop-augsburg.de; 6 Department of Obstetrics and Gynecology, MHH Hannover, 30625 Hannover, Germany; park-simon.tjoung-won@mh-hannover.de; 7 Department für Frauengesundheit, Universitäts-Frauenklinik Tübingen, 72076 Tübingen, Germany; eva-maria.grischke@med.uni-tuebingen.de; 8 Oncological Outpatient Department, Praxis für Hämatologie und Onkologie Koblenz, 56073 Koblenz, Germany; weide@onkologie-koblenz.de; 9 Department of Hematology/Oncology, Brüderkrankenhaus St. Josef Paderborn, 33098 Paderborn, Germany; h.mueller-huesmann@bk-paderborn.de; 10 Department of Hematology/Oncology, Niels Stensen Clinics, Franziskus Hospital, 49124 Georgsmarienhütte, Germany; kerstin.luedtke-heckenkamp@niels-stensen-kliniken.de; 11 Department of Gynaecology and Obstetrics, Hospital Ernst von Bergmann, 14467 Potsdam, Germany; dorothea.fischer@klinikumevb.de; 12 Department for Gynecology, Obstetrics and Reproductive Medicine, Saarland University Medical Center, 66421 Homburg, Germany; cosima.zemlin@uks.eu; 13 Klinikum Worms Frauenklinik, 67550 Worms, Germany; matthias.koegel@klinikum-worms.de; 14 Eisai Inc., Nutley, NJ 07110, USA; yan_jia@eisai.com; 15 Medical Department, Eisai GmbH, 60549 Frankfurt, Germany; helga_schmitz@eisai.net; 16 Medical Affairs, Eisai GmbH, 60549 Frankfurt, Germany; christian_engelbrecht@eisai.net; 17 Evangelische Kliniken Essen-Mitte gGmbH (KEM), 45136 Essen, Germany; c.jackisch@kem-med.com

**Keywords:** breast neoplasms, peripheral nervous system diseases, eribulin, cohort studies, prospective studies, medical oncology

## Abstract

Eribulin is a preferred treatment for patients with advanced breast cancer (BC) following anthracyclines and taxanes. The final analysis of the IRENE study assessed the incidence and resolution of eribulin-induced peripheral neuropathy (EIPN), along with safety and quality of life (QoL), in patients with advanced/metastatic BC. IRENE was an observational, single-arm, prospective, multicenter cohort study. Patients aged ≥18 years with locally advanced/metastatic BC that progressed after 1–3 prior chemotherapeutic regimens received eribulin and were monitored for new-onset or worsening EIPN. Secondary endpoints included time to disease progression, safety, and health-related QoL. In total, 108 (32.2%) out of 335 patients experienced EIPN; 18 (5.4%) experienced grade ≥3 EIPN. Median time to EIPN resolution (EIPN ended or returned to baseline) was 78.7 weeks (95% CI 77.1—not estimable). Median time to disease progression was 4.5 months (95% CI 3.9–5.5). Treatment-emergent adverse events (TEAEs) occurred in 322 (96.1%) patients; serious TEAEs occurred in 185 (55.2%) patients. Incidence and resolution rates of EIPN were comparable with existing evidence from previous trials. TEAEs were consistent with the established eribulin safety profile, with no new safety signals. Eribulin treatment did not appear to affect QoL, as measured by EQ-5D-3L and EQ-VAS, or patient-reported neuropathy symptoms, as measured by the PNQ.

## Simple Summary

Peripheral neuropathy is a common side effect of microtubule-targeting chemotherapy agents used in the treatment of breast cancer, including eribulin. The final analysis of the IRENE study investigated the incidence and resolution of eribulin-induced peripheral neuropathy (EIPN) in adults with locally advanced or metastatic breast cancer that progressed after one to three prior chemotherapeutic regimens. The incidence and resolution rates of EIPN were comparable to those reported in previous trials. Eribulin did not appear to impact patient-reported quality of life. Hence, the results of the IRENE study provide further evidence for the use of eribulin as a preferred treatment for patients with pretreated advanced or metastatic breast cancer.

## 1. Introduction

Eribulin, a halichondrin-class microtubule-dynamics inhibitor commonly used to treat breast cancer [1,2], is a preferred treatment for patients with advanced breast cancer after treatment with anthracyclines and taxanes [3,4]. Peripheral neuropathy is a well-known dose-limiting side effect of eribulin [5] that has been shown to worsen with higher cumulative doses of eribulin [6]; however, data assessing the persistence and resolution of chemotherapy-induced peripheral neuropathy are limited. Furthermore, the incidence of peripheral neuropathy in patients with breast cancer treated with eribulin appears to be higher than in patients with other solid tumors treated with eribulin [5]. The prespecified interim analysis of the prospective Incidence and Resolution of Eribulin-Induced Peripheral Neuropathy (IRENE) cohort study (data cut-off date: 1 July 2019) assessed the incidence and severity of peripheral neuropathy in patients with locally advanced or metastatic breast cancer treated with eribulin [7]. Eribulin-induced peripheral neuropathy (EIPN) was observed in 67 (32.4%) patients; grade ≥3 EIPN was observed in 12 (5.8%) patients (all events were grade 3). Dose modifications or delays due to EIPN occurred in only 3 (1.4%) patients, and eribulin was terminated as a result of EIPN in 6 (2.9%) patients. Median time to resolution of EIPN was not reached. Treatment-emergent adverse events (TEAEs) were reported in 195 (93.8%) patients (grade ≥3 TEAEs were observed in 62.0% of patients).

Results of the interim analysis of IRENE [7] were consistent with those reported in randomized phase 3 clinical trials of eribulin in patients with locally recurrent or metastatic breast cancer [8,9]. The EMBRACE trial [8], which compared eribulin and treatment of physician’s choice, showed a similar incidence of peripheral neuropathy among patients who received eribulin (35%). Similarly, the randomized phase 3 trial comparing single-agent eribulin and capecitabine monotherapies (Study 301) [9] showed a comparable rate of peripheral neuropathy in patients who received eribulin (27.4%). Notably, the peripheral neuropathy incidence rate observed in the IRENE interim analysis (32.4%) was also similar to that reported in an observational study of EIPN in Japanese patients with HER2-negative inoperable/recurrent breast cancer (28.1%) [6]. As IRENE was also an observational post-authorization study, the comparable rates of peripheral neuropathy shown among the IRENE interim analysis and both observational and randomized clinical trials are notable. Adverse events (AEs) observed in the IRENE study were consistent with the known safety profile of eribulin [10,11], and no new safety signals were noted. Additionally, the overall rates of AEs reported in EMBRACE (99%) [8] and Study 301 (94.1%) [9] were similar to the rates reported in the IRENE interim analysis (93.8%) [7].

Herein, we report the final analysis of the IRENE study, including the incidence and severity of peripheral neuropathy and safety. Additionally, patient-reported measures of peripheral neuropathy symptoms and health-related quality of life (HRQoL), which were not previously reported, are included in this final analysis of IRENE.

## 2. Patients and Methods

### 2.1. Study Design and Patients

The study design and inclusion/exclusion criteria have been described previously [7] and are briefly summarized below. The IRENE trial (Incidence and resolution of Eribulin-Induced Peripheral Neuropathy) was an observational, single-arm, prospective, multicenter cohort study. Patients ≥ 18 years of age with locally advanced or metastatic breast cancer and disease progression after one to three prior chemotherapeutic regimens for advanced disease were eligible to participate. The maximum number of prior chemotherapeutic regimens was extended per a protocol amendment (previously, a maximum of two therapies were allowed) to allow more patients to be enrolled. Patients with previous eribulin treatment or who had a contraindication according to the eribulin label were excluded from the study. All patients provided signed written informed consent before participation in this study. Patients were treated with eribulin according to the label, and data documentation occurred during routine visits according to clinical practice at each site. The estimated observation period per patient was anticipated to be approximately 15 months, and patients were followed for EIPN until death, resolution of EIPN, or return of EIPN to baseline level, whichever occurred first.

### 2.2. Endpoints

The primary objective of the IRENE study was to characterize the incidence, frequency, and resolution of EIPN in adults with locally advanced or metastatic breast cancer that has progressed after one to three chemotherapeutic regimens for advanced disease. The primary endpoints included the number and proportion of patients experiencing EIPN (regardless of severity), the severity of EIPN (as determined by Common Terminology Criteria for Adverse Events [CTCAE] grade version 4.0), the frequency of dose modifications or discontinuation of eribulin due to EIPN, time to eribulin treatment discontinuation due to EIPN, the frequency of resolution of EIPN (EIPN ended or returned to baseline, as determined by CTCAE grade version 4.0), the time to resolution of EIPN (time from onset or worsening from baseline to the date of resolution [defined as the cessation of EIPN or return to baseline]), as determined by CTCAE grade version 4.0, and therapeutic interventions (e.g., analgesics) used to treat EIPN. EIPN ratings were carried out by the treating physician. Secondary endpoints included time to disease progression (TDP; defined as time from the start of eribulin treatment to investigator assessment of disease progression) and the number and proportion of patients experiencing non-serious and serious AEs (SAEs). Additionally, patient-reported peripheral neuropathy symptoms and HRQoL, secondary endpoints reported only at this final analysis timepoint, were measured using the Patient Neurotoxicity Questionnaire (PNQ) and the EQ-5D-3L Questionnaire, respectively.

### 2.3. Statistical Methods

Unless otherwise specified, primary and secondary endpoints were analyzed for all enrolled patients who received at least one dose of eribulin (safety analysis set). IRENE was an observational study; thus, descriptive analyses were performed for most endpoints. Patients who experienced more than one episode of EIPN were counted only once. Descriptive subgroup analyses based on age (<65 vs. ≥65 years) were performed for all primary endpoints.

Associations between the occurrence of new or worsening EIPN and peripheral neuropathy predispositions and between the occurrence of new or worsening PN and the existence of previous peripheral neuropathy were investigated with regression models. Univariate and multivariate logistic regression models including predisposition factors as independent variables were performed. A univariate logistic regression model served as a sensitivity analysis to assess the effect of the presence of past or pre-existing peripheral neuropathy on the probability of developing EIPN. The dependent variable was patients with at least one EIPN event versus patients without any EIPN events and given as a function of the existence of past or pre-existing peripheral neuropathy as documented at baseline.

Kaplan–Meier estimates of the time to onset of EIPN were performed for all patients in the safety analysis set. Descriptive statistics for the time to onset of first EIPN were also calculated based on the subset of patients with at least one EIPN event and for patients with resolution of all EIPN events. Kaplan–Meier estimates of time to resolution of EIPN were summarized and analyzed based on the subset of patients with at least one EIPN event. Descriptive statistics for time to resolution of EIPN were also calculated based on the subset of patients with resolution of all ongoing EIPN events (in case of worsening EIPN, resolution was defined as EIPN returned to baseline status). Time to termination of eribulin treatment due to EIPN was estimated using Kaplan–Meier methodology based on the subset of patients with at least one EIPN event. Time to improvement of EIPN (either resolution of the EIPN or a decrease in EIPN grade) based on the presence or absence of therapeutic intervention used to treat EIPN was analyzed descriptively and using the Kaplan–Meier method, based on patients with at least one EIPN event. Time to improvement of EIPN by presence and absence of therapeutic intervention used to treat EIPN was also analyzed descriptively, based on the subset of patients with improvement of all EIPN events (either resolution of the EIPN or a decrease in EIPN grade).

TDP was analyzed and plotted for all patients in the safety analysis set using Kaplan–Meier methodology. The analysis was repeated for patients with and without EIPN. For HRQoL measures, descriptive statistics by treatment cycle were provided for PNQ score, EQ-5D total score, visual analogue scale (VAS), and the respective changes from baseline. Numbers and percentages of AEs and treatment-related AEs by preferred term and CTCAE v4.0 grade were reported descriptively. Numbers and percentages of SAEs and treatment-related SAEs were also reported descriptively.

## 3. Results

### 3.1. Patients

At the data cutoff date for the final analysis (1 June 2022), 335 patients who received at least one dose of eribulin were included in the safety analysis set. A total of 313 (93.4%) patients terminated eribulin treatment (the main reasons for eribulin termination were disease progression [*n* = 200; 59.7%] and AEs [*n* = 57; 17.0%]); a total of 330 (98.5%) patients terminated the study. Aside from regular termination of eribulin treatment (*n* = 141; 42.1%), the main reasons for study termination were death (*n* = 79; 23.6%), AEs (*n* = 32; 9.6%), and withdrawal of informed consent (*n* = 10; 3.0%). Patient disposition is displayed in Figure S1.

Patient demographics and clinical characteristics at baseline are shown in Table 1. Of 335 total patients, 301 (89.9%) had previously received neurotoxic anticancer treatment, 113 (33.7%) had a predisposition to peripheral neuropathy, and 147 (43.9%) had peripheral neuropathy ongoing at baseline. Of the 147 patients with peripheral neuropathy at baseline, the majority (73.5%) had a maximum peripheral neuropathy grade of 1.

Peripheral neuropathy incidence by preferred term is shown in Table S1. EIPN incidence and resolution, therapeutic interventions for EIPN, and EIPN events leading to dose modification, delay, or termination are summarized in Table 2. Of 335 total patients, 108 (32.2%) experienced any-grade EIPN, and 18 (5.4%) experienced grade ≥3 EIPN. Worsening of pre-existing peripheral neuropathy occurred in 53 (15.8%) patients, and 82 (24.5%) patients had new-onset peripheral neuropathy. Results of the sensitivity analysis with univariate logistic regression analysis indicated no correlation between pre-existing peripheral neuropathy and the incidence of EIPN. The relationship between peripheral neuropathy predispositions and the incidence of EIPN was also analyzed using logistic regression analyses. None of the predisposition factors provided enough information to explain the occurrence of EIPN (by means of the univariate or multivariate models).

**Figure 1:**
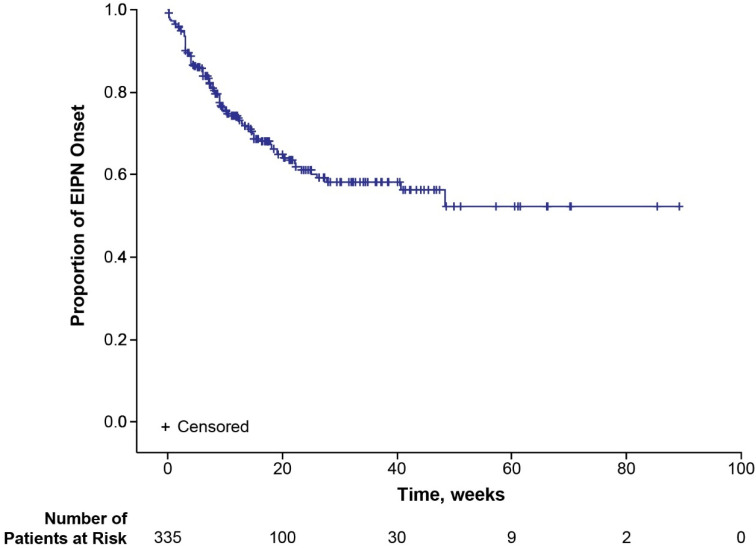
Kaplan–Meier plot for time to first onset of EIPN. EIPN, eribulin-induced peripheral neuropathy.

**Figure 2:**
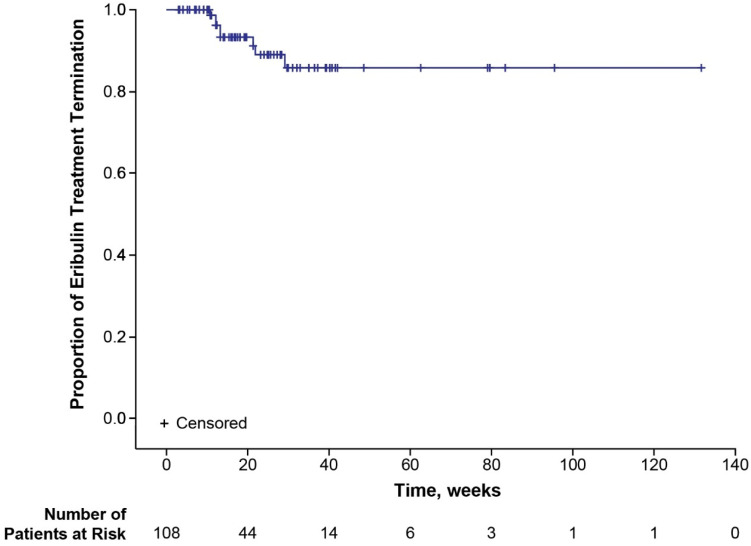
Kaplan–Meier plot for time to eribulin treatment termination due to EIPN. EIPN, eribulin-induced peripheral neuropathy.

**Figure 3:**
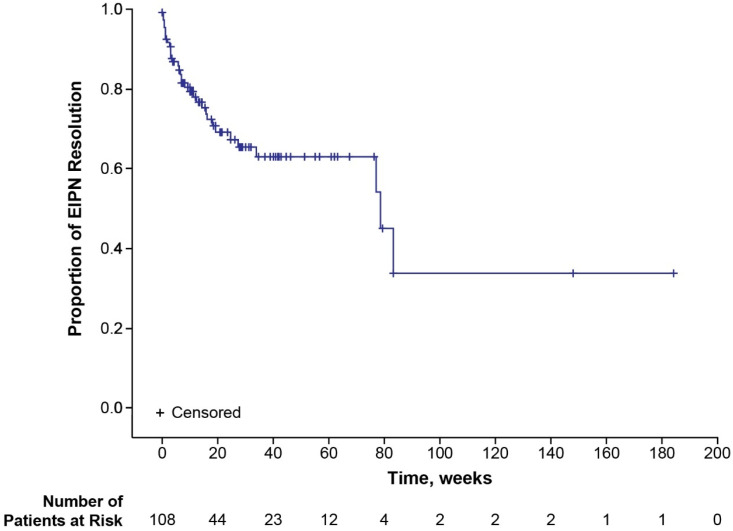
Kaplan–Meier plot for time to resolution of EIPN. EIPN, eribulin-induced peripheral neuropathy.

Median time to onset of first EIPN was not reached (95% CI 40.7—not estimable (NE)) in the total population (safety analysis set) (Figure 1). When examined by age group, median time to onset of first EIPN was not reached (95% CI NE-NE) in patients <65 years of age and 48.4 weeks (95% CI 18.1-NE) in patients ≥65 years of age. Modifications, delays, and terminations of eribulin due to EIPN were uncommon (Table 2). Eight (2.4%) patients terminated eribulin treatment due to EIPN. Median time to eribulin treatment termination due to EIPN was not reached for the total population (Figure 2) nor for the subgroups of younger (<65 years of age) or older (≥65 years of age) patients. Of the 108 patients with EIPN, 34 (31.5%) experienced resolution of EIPN (defined as all ongoing EIPN events resolved or, in the case of worsening EIPN, returned to baseline levels) (Table 2). A higher percentage of younger (<65 years) patients experienced EIPN resolution than older (≥65 years) patients (37.5% vs. 22.7%). Median time to resolution of EIPN in the total population was 78.7 weeks (95% CI 77.1-NE) (Figure 3) and comparable in younger (<65 years) patients (77.1 weeks [95% CI 18.3-NE]). Median time to resolution of EIPN was not reached (95% CI 83.1-NE) in older (≥65 years) patients. Times to onset and resolution of EIPN in the subset of patients with EIPN events are reported in Table S2.

Of the 108 patients with EIPN events, therapeutic interventions for EIPN were used in 21 (19.4%) patients. The most common interventions were pregabalin (*n* = 9), vitamin B12 (*n* = 5), and pyridoxine hydrochloride (*n* = 5) (Table 2). Of the 21 patients who were given therapeutic interventions to treat EIPN, five (23.8%) patients experienced EIPN improvement. Median time to improvement was 77.1 weeks (95% CI 77.1–78.7) based on the Kaplan–Meier estimate (Figure S2). Of the 87 patients with EIPN who did not receive a therapeutic intervention, 31 (35.6%) patients achieved improvement. Median time to improvement without therapeutic intervention was 83.1 weeks (95% CI 24.7-NE) based on the Kaplan–Meier estimate (Figure S2). Times to improvement of EIPN in the subset of patients with improvement of all EIPN events (based on the presence or absence of therapeutic intervention) are reported in Table S2.

### 3.2. Time to Disease Progression

**Figure 4:**
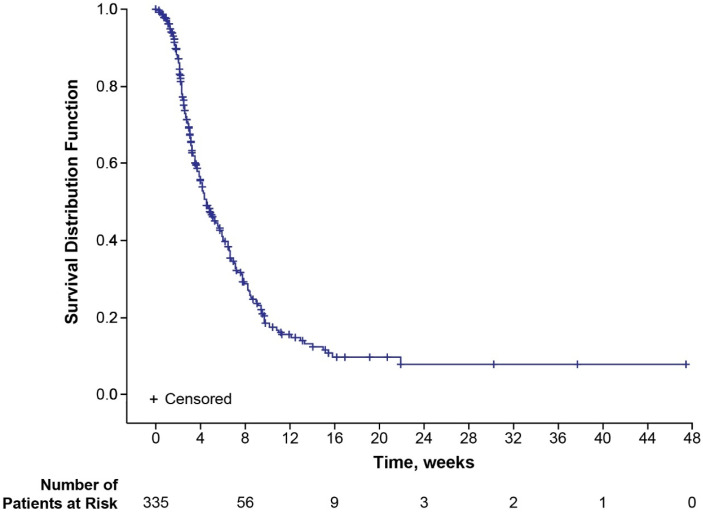
Kaplan–Meier plot for time to disease progression.

Over the course of the study, 221 (66.0%) patients experienced disease progression. The median TDP was 4.5 months (95% CI 3.9–5.5) (Figure 4). In the subset of patients with EIPN (108 patients), 66 (61.1%) patients had disease progression; the median TDP was 6.5 months (95% CI 4.6–7.8) based on the Kaplan–Meier estimate (Figure S3). For patients without EIPN (*n* = 227 patients), 155 (68.3%) had disease progression; the median TDP for this subset of patients was 3.9 months (95% CI 3.3–4.8) based on the Kaplan–Meier estimate (Figure S3).

### 3.3. Safety

TEAEs are summarized in Table 3. TEAEs of any grade and grade ≥ 3 occurred in 322 (96.1%) and 214 (63.9%) patients, respectively. Non-fatal and fatal serious TEAEs occurred in 139 (41.5%) and 81 (24.2%) patients, respectively. Notably, 60 (17.9%) patients in the safety population died of malignant neoplasm progression or another malignant neoplasm-associated event and were included in the fatal serious TEAE count, as worsening of underlying malignant disease was reported as a TEAE until protocol amendment v1.1. After this amendment, 34 additional patients died due to disease progression, and these events were not documented as AEs. Eribulin-related TEAEs occurred in 238 (71.0%) patients and serious eribulin-related TEAEs occurred in 57 (17.0%) patients. Eribulin-related TEAEs led to dose modifications in 52 (15.5%) patients and eribulin termination in 16 (4.8%) patients. Similar rates of overall and eribulin-related TEAEs were observed in younger (<65 years) and older (≥65 years) patients (Table 3). The most common TEAEs that were judged by the investigator to be at least possibly related to eribulin were leukopenia and fatigue (*n* = 55 [16.4%] each), peripheral sensory neuropathy (*n* = 49 [14.6%]), neutropenia ([*n* = 45 [13.4%]), and alopecia ([*n* = 44] (13.1%) (Table S3).

### 3.4. Patient-Reported Outcomes

Results of the PNQ displayed up to cycle 5 (which corresponds to the median eribulin treatment duration) are shown in Table S4. At baseline, 286 (85.4%) patients completed the PNQ. Of the patients with available PNQ data, most reported no (32.9%) or mild (38.1%) symptoms of “numbness, pain or tingling in hands and feet” (Item 1), while 15.7% of patients had moderate symptoms, 12.2% had moderate-to-severe symptoms, and 1.0% had severe symptoms. At cycle 5, there was a trend toward more mild or moderate symptoms (Item 1: mild, 48.4%; moderate, 22.7%), while moderate-to-severe and severe symptoms were similar to baseline levels. For Item 2 (“weakness in arms and legs”), symptoms after five cycles were comparable to those at baseline. Per the results of the EQ-5D-3L and EQ-VAS questionnaires, eribulin treatment did not appear to negatively impact QoL or health-state scores (Tables S5 and S6).

A subgroup analysis of the PNQ in patients with EIPN is shown in Table S7. At baseline, 96 of 108 patients (88.8%) completed a PNQ. Most patients reported having no (40.6%) or mild (34.4%) symptoms of “numbness, pain or tingling in hands and feet” (Item 1), while 12.5% of patients had moderate-to-severe or severe symptoms. At cycle 5, the proportion of patients without symptoms decreased (20.0%), while the proportion of patients with mild or moderate symptoms and moderate-to-severe symptoms increased (mild 41.7%; moderate 23.3%; moderate-to-severe 15.0%). For Item 2 (“weakness in arms and legs”), most patients reported no (37.1%), mild (24.7%), or moderate (16.5%) symptoms at baseline, while 21.7% had moderate-to-severe, or severe symptoms. After 5 cycles, the proportion of patients with moderate-to-severe or severe symptoms remained consistent (22.4%), while the proportion of patients with no symptoms decreased (24.1%) and the proportion of patients with mild or moderate symptoms increased compared with those at baseline (mild: 32.8%, moderate 20.7%).

## 4. Discussion

The purpose of the IRENE study was to characterize the incidence and resolution of EIPN in patients with advanced or metastatic breast cancer, as information regarding the nature of this commonly occurring side effect of microtubule-targeting chemotherapy is limited. The EIPN incidence profile observed in the final analysis of IRENE was similar to that observed in the interim analysis [7]. Compared with the results of the European Medicine Agency’s (EMA’s) Halaven assessment report [12], which pooled results from seven phase 2/3 clinical trials in patients with advanced or metastatic breast cancer who were treated with eribulin (for a total pooled safety population of 1503 patients), patients in the IRENE study had slightly lower incidences of any-grade EIPN (36% vs. 32.2%). The incidence of grade ≥3 EIPN in the final analysis of the IRENE study (5.4%) was also lower than that of the EMA’s Halaven assessment report (7.7%) [12]. In the IRENE study, EIPN of any grade was numerically higher in older (≥65 years) versus younger (<65 years) patients (36.4% vs. 29.9%). The frequency of eribulin treatment termination due to EIPN (2.4%) was slightly lower than in the Halaven assessment report (3.3%) [12]. Notably, the characterization of peripheral neuropathy in the Halaven assessment report [12] was based on an earlier version of the MedDRA; thus, comparisons must be approached with caution.

The EIPN resolution rate observed in the final analysis of IRENE (31.5%) was comparable with that reported in the Halaven assessment report (27.7%); however, the resolution analysis in the Halaven assessment report was based on narrow Standardized MedDRA Queries, so not all preferred terms were included [12]. Median time to resolution of EIPN, which was not reached at the interim analysis [7], was considerably longer than the median time to resolution reported in the Halaven assessment report (78.7 vs. 8.1 weeks); notably, this discrepancy is due to the IRENE data set being analyzed using Kaplan-Meyer estimates, with 108 of 335 patients having the event ‘resolution of EIPN.’ In the subset of patients who had resolution of all EIPN events, the median (6.71 weeks) was comparable to the results of the assessment report. Notably, the high percentage of censored patients (68.5%) due to death (31.5%) and start of another neurotoxic anticancer therapy (20.4%) in the Kaplan–Meier analysis of time to resolution of EIPN affected the time to resolution analysis.

In contrast to the Halaven assessment report [12], fewer patients in the IRENE study experienced dose modifications (22.7% vs. 50.7%) or dose reductions (20.3% vs. 30.9%), while dose delays were higher in the IRENE study (73.4% vs. 40.8%). The overall incidence of TEAEs, including eribulin-related TEAEs, was similar to that observed in the interim analysis; no new safety signals were recorded, and TEAEs were consistent with eribulin’s known safety profile [10]. While the occurrence of grade ≥3 TEAEs was slightly lower in this study versus the Halaven assessment report (63.9% vs. 70.9%), the occurrence of SAEs (55.2% vs. 23.4%) and fatal SAEs (24.2% vs. 4.5%) was higher. However, most of the fatal SAEs were due to malignant neoplasm progression, which was considered a TEAE until the first protocol amendment. The percentage of patients who experienced eribulin treatment termination due to a TEAE was lower in IRENE (4.8%) than in the Halaven assessment report (10.4%) [12].

As shown in the interim analysis [7], the overall rates of EIPN and TEAEs observed in the IRENE final analysis were also similar to that of the individual EMBRACE [8] and Study 301 [9] randomized phase 3 trials, two of the trials incorporated into the pooled analysis conducted in the Halaven assessment report [12]. Similar rates of any-grade EIPN were reported in the final analysis of IRENE (32.2%) compared to the peripheral neuropathy rates observed in EMBRACE (35%) and Study 301 (27.4%). Similar to the IRENE final analysis, where 96.1% of patients experienced any-grade TEAEs, the majority of patients treated with eribulin in these trials experienced any-grade TEAEs (99% in EMBRACE and 94.1% in Study 301).

Eribulin treatment did not appear to affect QoL as measured using EQ-5D-3L and EQ-VAS, or patient-reported neuropathy symptoms, as measured by the PNQ. This outcome is clinically significant and provides further support for eribulin as a preferred chemotherapy in patients with pretreated advanced/metastatic breast cancer. However, the results of these analyses must be interpreted with caution, as many questionnaires for each measure were missing. Future studies are needed to confirm the impact of EIPN on quality of life and to explore the relationship between physician-rated EIPN and patient reports of peripheral neuropathy symptoms. Additionally, while a more detailed characterization of quality of life in subgroups of patients (e.g., those who experienced EIPN of varying intensities or those who received versus did not receive therapeutic interventions for peripheral neuropathy) was beyond the scope of the IRENE study; a more fine-grained approach in future studies would further elucidate the impact of EIPN on patient-reported quality of life.

## 5. Conclusions

Together with the results of the interim analysis [7], the results of this final analysis of the IRENE study signal the importance of monitoring peripheral neuropathy during eribulin treatment and add to the limited body of work on the characterization of EIPN in patients with advanced/metastatic breast cancer treated in real-world settings. Additional studies, particularly those that focus on patient-reported QoL measures, are warranted to further characterize EIPN in this patient population.

## Figures and Tables

**Table 1:** Baseline patient demographics and clinical characteristics.

Characteristic	Patients ^a^ (*n* = 335)
**Age, years** Median (range) Mean (SD)	60.0 (32.0, 83.0) 60.0 (11.2)
**Sex, *n* (%)** Female Male	334 (99.7) 1 (0.3)
**Mean body weight, kg (SD) ^b^**	71.2 (16.4)
**Locally advanced breast cancer, *n* (%)**	17 (5.1)
**Metastatic sites, *n* (%)** Bone Brain Lung Liver Other Missing	318 (94.9) 219 (65.4) 28 (8.4) 134 (40.0) 178 (53.1) 148 (44.2) 0
**Subtype, *n* (%)** Luminal A Luminal B HER2-enriched Basal-like Missing	56 (16.7) 86 (25.7) 39 (11.6) 46 (13.7) 108 (32.2)
**Patients with ≥ 1 previous anticancer treatment, *n* (%)** 1 2 ≥3	330 (98.5) 15 (4.5) 26 (7.8) 289 (86.3)
**Type of previous anticancer treatment, *n* (%) ^c^** Paclitaxel Cyclophosphamide Epirubicin Bevacizumab Fulvestrant Capecitabine Letrozole Tamoxifen Docetaxel Palbociclib	330 (98.5) 228 (68.1) 177 (52.8) 153 (45.7) 131 (39.1) 104 (31.0) 99 (29.6) 92 (27.5) 83 (24.8) 82 (24.5) 75 (22.4)
**Previous neurotoxic anticancer treatment, *n* (%)** Taxanes Platin derivatives Vinca alkaloids Other	301 (89.9) 294 (87.8) 68 (20.3) 19 (5.7) 19 (5.7)
**Predisposition for peripheral neuropathy, *n* (%)** Hypothyreosis Diabetes mellitus type 1 or 2 Renal impairment Inflammatory diseases Herpes zoster Alcohol abuse Other	113 (33.7) 58 (17.3) 33 (9.9) 15 (4.5) 11 (3.3) 7 (2.1) 1 (0.3) 9 (2.7)
**Peripheral neuropathy ongoing at baseline, *n* (%)** **Maximum CTCAE grade, *n* ^d^** 1 2 3	147 (43.9) 108 (32.2) 69 (20.6) 6 (1.8)

^a^ Percentages in this column are based on the total number of patients (*n* = 335); ^b^ nine patients had missing data for this parameter; ^c^ previous anticancer therapies received by >20% of patients are shown; ^d^ grade information was missing for two patients. CTCAE, Common Terminology Criteria for Adverse Events; HER2, human epidermal growth factor receptor 2; SD, standard deviation.

**Table 2:** Patients who experienced EIPN.

Incidence, *n* (%)	Total (*n* = 335)	Age <65 Years (*n* = 214)	Age ≥65 Years (*n* = 121)
Any EIPN event ^a^ Worsening of pre-existing peripheral neuropathy New-onset EIPN	108 (32.2) 53 (15.8) 82 (24.5)	64 (29.9) 31 (14.5) 51 (23.8)	44 (36.4) 22 (18.2) 31 (25.6)
Any EIPN event of grade ≥3	18 (5.4)	12 (5.6)	6 (5.0)
Resolution of all EIPN events ^b,c^	34 (31.5)	24 (37.5)	10 (22.7)
Any therapeutic intervention for EIPN ^c,d^	21 (19.4)	13 (20.3)	8 (18.2)
Any EIPN event leading to dose modification ^e^	13 (3.9)	6 (2.8)	7 (5.8)
Any EIPN event leading to dose delay ^f^	5 (1.5)	2 (0.9)	3 (2.5)
Any EIPN event leading to eribulin termination	8 (2.4)	6 (2.8)	2 (1.7)

^a^ Patients appear in each category in which they had ≥ one event; ^b^ resolution is defined as all ongoing EIPN events resolved or, in the case of worsening EIPN, returned to baseline levels; ^c^ percentages are based on the number of patients with any EIPN event; ^d^ the most common interventions were pregabalin (*n* = 9), vitamin B12 (*n* = 5), and pyridoxine hydrochloride (*n* = 5); ^e^ a dose modification is defined as a change in administered dose between two study visits; ^f^ a dose delay is defined as a period of more than seven days between day 1 and day 8 within a treatment cycle or as a period of > 14 days between day 8 and day 1 of the following treatment cycle. EIPN, eribulin-induced peripheral neuropathy.

**Table 3:** Summary of TEAEs.

TEAEs, *n* (%)	Total (*n* = 335)	Age < 65 Years (*n* = 214)	Age ≥ 65 Years (*n* = 121)
Any TEAE	322 (96.1)	208 (97.2)	114 (94.2)
Eribulin-related TEAE	238 (71.0)	149 (69.6)	89 (73.6)
TEAE grade ≥ 3	214 (63.9)	141 (65.9)	73 (60.3)
Serious TEAE Non-fatal Fatal ^a,b^	185 (55.2) 139 (41.5) 81 (24.2)	122 (57.0) 93 (43.5) 54 (25.2)	63 (52.1) 46 (38.0) 27 (22.3)
Serious eribulin-related TEAE	57 (17.0)	37 (17.3)	20 (16.5)
Eribulin-related TEAEs leading to dose modification	52 (15.5)	34 (15.9)	18 (14.9)
Eribulin-related TEAEs leading to eribulin termination	16 (4.8)	9 (4.2)	7 (5.8)

^a^ Of the patients who had fatal serious TEAEs, five (1.5%) patients had treatment-related grade 5 events at least possibly related to eribulin. They included infections and infestations (*n* = 2), general physical health deterioration, stomatitis, and dyspnea (*n* = 1 each). AEs were documented in a way that allowed three different terms to be linked to a single event. As a result, stomatitis and dyspnea may appear as grade 5 because investigators linked them as such, even though according to the CTCAE v4.0, grade 5 events are not possible; ^b^ sixty (17.9%) patients in the safety population died of malignant neoplasm progression or another malignant neoplasm-associated event and were included in the fatal serious TEAE count, as worsening of underlying malignant disease was reported as a TEAE until protocol amendment v1.1. Thirty-four further patients died due to disease progression after this amendment, and these events were not documented as AEs. AE, adverse event; CTCAE v4.0, Common Terminology Criteria for Adverse Events version 4.0; TEAE, treatment-emergent adverse event.

## Data Availability

The data are not available for sharing at this time because the data are commercially confidential. However, Eisai Inc. will consider written requests to share the data on a case-by-case basis.

## Supplementary Materials

The following supporting information can be downloaded at: https://www.mdpi.com/article/10.3390/cancers17030457/s1, Figure S1: Patient disposition; Figure S2: Kaplan–Meier plot for time to improvement of EIPN by the presence (red line) or absence (blue line) of therapeutic intervention; Figure S3: Kaplan–Meier plot for time to disease progression by the presence (red line) or absence (blue line) of EIPN; Table S1: Time to onset, resolution, and improvement of EIPN in subsets of patients with EIPN events, improvement, or resolution; Table S2: Most common eribulin-related TEAEs (>5% any grade); Table S3: Patient Neurotoxic Questionnaire (total population); Table S4: Patient Neurotoxic Questionnaire (total population); Table S5: Results of the EQ-5D-3L; Table S6: EQ-VAS health state scores; Table S7: Patient Neurotoxic Questionnaire (patients with EIPN).

## References

[1] Cardoso F., Paluch-Shimon S., Senkus E., Curigliano G., Aapro M.S., André F., Barrios C.H., Bergh J., Bhattacharyya G.S., Biganzoli L. (2020). 5th ESO-ESMO international consensus guidelines for advanced breast cancer (ABC 5). Ann. Oncol..

[2] Vahdat L.T., Garcia A.A., Vogel C., Pellegrino C., Lindquist D.L., Iannotti N., Gopalakrishna P., Sparano J.A. (2013). Eribulin mesylate versus ixabepilone in patients with metastatic breast cancer: A randomized Phase II study comparing the incidence of peripheral neuropathy. Breast Cancer Res. Treat..

[3] Gennari A., Andre F., Barrios C.H., Cortes J., de Azambuja E., DeMichele A., Dent R., Fenlon D., Gligorov J., Hurvitz S.A. (2021). ESMO Clinical Practice Guideline for the diagnosis, staging and treatment of patients with metastatic breast cancer. Ann. Oncol..

[4] National Comprehensive Cancer Network Clinical Practice Guidelines in Oncology (NCCN Guidelines®) Breast Cancer. Version 6.2024. https://www.nccn.org/professionals/physician_gls/pdf/breast.pdf.

[5] Peng L., Hong Y., Ye X., Shi P., Zhang J., Wang Y., Zhao Q. (2017). Incidence and relative risk of peripheral neuropathy in cancer patients treated with eribulin: A meta-analysis. Oncotarget.

[6] Tsurutani J., Sakata Y., Matsuoka T. (2019). Chemotherapy-induced peripheral neuropathy in breast cancer patients treated with eribulin: Interim data from a post-marketing observational study. Breast Cancer.

[7] Lück H.J., Schmidt M., Hesse T., Hoffmann O., Heinrich B.J., Park-Simon T.W., Grischke E.M., Weide R., Müller-Huesmann H., Lüdtke-Heckenkamp K. (2023). Incidence and Resolution of Eribulin-Induced Peripheral Neuropathy (IRENE) in locally advanced or metastatic breast cancer: Prospective cohort study. Oncologist.

[8] Cortes J., O’Shaughnessy J., Loesch D., Blum J.L., Vahdat L.T., Petrakova K., Chollet P., Manikas A., Diéras V., Delozier T. (2011). Eribulin monotherapy versus treatment of physician’s choice in patients with metastatic breast cancer (EMBRACE): A phase 3 open-label randomised study. Lancet.

[9] Kaufman P.A., Awada A., Twelves C., Yelle L., Perez E.A., Velikova G., Olivo M.S., He Y., Dutcus C.E., Cortes J. (2015). Phase III open-label randomized study of eribulin mesylate versus capecitabine in patients with locally advanced or metastatic breast cancer previously treated with an anthracycline and a taxane. J. Clin. Oncol.

[10] Eisai Inc (2022). Halaven (Eribulin mesylate) [Package Insert].

[11] Eisai Europe Ltd (2017). Halaven 0.44 mg/mL [Fachinformation].

[12] European Medicines Agency Halaven: EPAR Assessment Report. EMA/441074/2014. Procedure No.: EMEA/H/C/002084/II/0011. https://www.ema.europa.eu/en/documents/variation-report/halaven-h-c-2084-ii-0011-epar-assessment-report-variation_en.pdf.

