# Supplementary material for: Eribulin-Induced Peripheral Neuropathy in Locally Advanced or Metastatic Breast Cancer: Final Analysis of the Prospective Cohort IRENE Study
Source: Cancers (Basel). 2025 Jan 28;17(3):457. doi: 10.3390/cancers17030457 (PMC11815746; doi:10.3390/cancers17030457)
Supplement: Supplementary file 1 [file cancers-17-00457-s001.zip › cancers-3366292-supplementary.pdf]

Table S1. Patients[a] with peripheral neuropathy (by preferred term).

Preferred term, n (%)	Any grade	Grade ≥3
Patients with at least one peripheral neuropathy event	108 (100)	18 (16.7)
Peripheral sensory neuropathy	52 (48.1)	10 (9.3)
Paraesthesia	22 (20.4)	2 (1.9)
Hypoesthesia	20 (18.5)	4 (3.7)
Peripheral sensorimotor neuropathy	13 (12.0)	3 (2.8)
Muscular weakness	7 (6.5)	1 (0.9)
Polyneuropathy	5 (4.6)	1 (0.9)
Neuralgia	3 (2.8)	1 (0.9)
Peripheral motor neuropathy	3 (2.8)	1 (0.9)
Muscle spasms	2 (1.9)	0
Dysgeusia	1 (0.9)	0
Neuropathy peripheral	1 (0.9)	0
Taste disorder	1 (0.9)	0

Percentages are based on the number of patients with at least one peripheral neuropathy event. Peripheral neuropathy events were graded according to the CTCAE v4.0 and coded according to MedDRA v25.0. A patient appears in each category in which he had at least one event but only the most severe event is counted if the patient reported more than one event in the same category.

[a]The total population (all treated patients) was 335.

CTCAE v4.0, Common Terminology Criteria for Adverse Events version 4.0; MedDRA v25.0, Medical Dictionary for Drug Regulatory Activities version 25.0.

Table S2. Time to onset, resolution, and improvement of EIPN in subsets of patients with EIPN events, improvement, or resolution[a].

	**Total (N = 355)**	**< 65 years of age (n = 214)**	**≥ 65 years of age (n = 121)**
Time to onset of first EIPN (in patients with at least one EIPN), weeks			
Patients, n	108	64	44
Median (range)	7.21 (0.1, 48.4)	6.64 (0.1, 27.7)	7.71 (0.1, 48.4)
Time to onset of first EIPN (in patients with resolution of all EIPNs), *weeks*			
Patients, n	34	24	10
Median (range)	4.14 (0.1, 27.7)	4.29 (0.1, 27.7)	3.64 (0.3, 18.1)
Time to resolution of all EIPNs (in patients with resolution of all EIPNs), weeks			
Resolved EIPNs, n	50	39	11
Median (range)	6.71 (0.1, 83.1)	9.14 (0.1, 78.7)	3.14 (0.4, 83.1)
Time to improvement of EIPN with presence of therapeutic intervention (in patients with improvement of all EIPNs), weeks[b]			
Patients, n	8	NA	NA
Median (range)	44.71 (1.1, 78.7)	NA	NA
Time to improvement of EIPN with absence of therapeutic intervention (in patients with improvement of all EIPNs), weeks[b]			
Patients, n	44	NA	NA
Median (range)	6.43 (0.1, 83.1)	NA	NA

[a]Analyzed using descriptive statistics; [b]an analysis by age was not carried out for these subgroups.

EIPN, eribulin-induced peripheral neuropathy; NA, not available.

Table S3. Most common eribulin-related TEAEs (> 5% any grade)[a].

Adverse Events, n (%)	Total (N = 335)			
	**Any Grade**	**Grade 3**	**Grade 4**	**Grade 5**
Leukopenia	55 (16.4)	27 (8.1)	5 (1.5)	0
Fatigue	55 (16.4)	11 (3.3)	0	0
Peripheral sensory neuropathy	49 (14.6)	8 (2.4)	0	0
Neutropenia	45 (13.4)	18 (5.4)	6 (1.8)	0
Alopecia	44 (13.1)	5 (1.5)	0	0
Nausea	26 (7.8)	2 (0.6)	0	0
Stomatitis	20 (6.0)	1 (0.3)	0	1 (0.3)[b]
Paresthesia	19 (5.7)	2 (0.6)	0	0
Anemia	19 (5.7)	6 (1.8)	2 (0.6)	0
Hypoesthesia	18 (5.4)	4 (1.2)	0	0
Constipation	17 (5.1)	0	0	0

[a]TEAEs reported as at least possibly related to eribulin are included. Severity was graded according to CTCAE version 4.0. At each level of patient summarization, only the most-severe event was counted if the patient reported more than one event. AEs were coded with the MedDRA version 25.0; [b]AEs were documented in a way that allowed three different terms to be linked to a single event. As a result, these events may appear as grade 5 events because investigators linked them as such, even though according to CTCAE, certain grade 5 events are not possible. AE, adverse event; CTCAE, Common Terminology Criteria for Adverse Events; MedDRA, Medical Dictionary for Regulatory Activities; TEAE, treatment-emergent adverse event.

Table S4. Patient Neurotoxic Questionnaire (total population).

Cycle	Total Population (N = 335)					
	A[a] no	B[a] mild	C[a] moderate	D[a] moderate to severe	E[a] severe	Missing[b]
	Item 1: Numbness, pain or tingling in hands and feet, n (%)					
Baseline	94 (32.9)	109 (38.1)	45 (15.7)	35 (12.2)	3 (1.0)	49 (14.6)
1	15 (20.3)	36 (48.6)	8 (10.8)	13 (17.6)	2 (2.7)	261 (77.9)
2	69 (30.4)	95 (41.9)	38 (16.7)	23 (10.1)	2 (0.9)	108 (32.2)
3	51 (26.0)	93 (47.4)	31 (15.8)	17 (8.7)	4 (2.0)	139 (41.5)
4	34 (21.7)	67 (42.7)	31 (19.7)	23 (14.6)	2 (1.3)	178 (53.1)
5	24 (18.8)	62 (48.4)	29 (22.7)	13 (10.2)	0	207 (61.8)
	Item 2: Weakness in arms or legs, n (%)					
Baseline	88 (30.8)	91 (31.8)	48 (16.8)	54 (18.9)	5 (1.7)	49 (14.6)
1	17 (23.6)	24 (33.3)	13 (18.1)	15 (20.8)	3 (4.2)	263 (78.5)
2	60 (26.4)	88 (38.8)	38 (16.7)	39 (17.2)	2 (0.9)	108 (32.2)
3	49 (25.0)	79 (40.3)	30 (15.3)	34 (17.3)	4 (2.0)	139 (41.5)
4	42 (26.9)	51 (32.7)	29 (18.6)	31 (19.9)	3 (1.9)	179 (53.4)
5	32 (25.2)	50 (39.4)	25 (19.7)	19 (15.0)	1 (0.8)	208 (62.1)

[a]Percentages are based on the number of nonmissing questionnaires; [b]percentages are based on the total population.

Table S5. Results of the EQ-5D-3L

	Total population (N = 335)			
Cycle	1<br>no problems	2<br>some problems	3<br>extreme problems	Missing
	**Mobility, n (%)**			
Baseline	182 (63.9)	102 (35.8)	1 (0.4)	50
1	46 (63.9)	26 (36.1)	0	263
2	131 (58.0)	95 (42.0)	0	109
3	104 (53.3)	90 (46.2)	1 (0.5)	140
4	96 (60.0)	64 (40.0)	0	175
5	82 (64.1)	46 (35.9)	0	207
	**Self-care, n (%)**			
Baseline	231 (81.1)	52 (18.2)	2 (0.7)	50
1	54 (76.1)	15 (21.1)	2 (2.8)	264
2	177 (79.4)	42 (18.8)	4 (1.8)	112
3	150 (77.7)	40 (20.7)	3 (1.6)	142
4	129 (80.6)	30 (18.8)	1 (0.6)	175
5	112 (86.2)	15 (11.5)	3 (2.3)	205
	**Usual activities, n (%)**			
Baseline	114 (39.7)	151 (52.6)	22 (7.7)	48
1	31 (43.1)	34 (47.2)	7 (9.7)	263
2	92 (40.4)	124 (54.4)	12 (5.3)	107
3	85 (43.8)	102 (52.6)	7 (3.6)	141
4	75 (46.9)	77 (48.1)	8 (5.0)	175
5	65 (50.0)	62 (47.7)	3 (2.3)	205
	**Pain/discomfort, n (%)**			
Baseline	85 (29.5)	186 (64.6)	17 (5.9)	47
1	14 (19.4)	49 (68.1)	9 (12.5)	263
2	64 (28.3)	147 (65.0)	15 (6.6)	109
3	56 (28.6)	125 (63.8)	15 (7.7)	139
4	45 (28.3)	99 (62.3)	15 (9.4)	176
5	39 (30.5)	83 (64.8)	6 (4.7)	207
	**Anxiety/depression, n (%)**			
Baseline	131 (45.6)	143 (49.8)	13 (4.5)	48
1	27 (37.5)	39 (54.2)	6 (8.3)	263
2	93 (41.5)	123 (54.9)	8 (3.6)	111
3	88 (45.4)	97 (50.0)	9 (4.6)	141
4	79 (50.0)	73 (46.2)	6 (3.8)	177
5	74 (57.8)	53 (41.4)	1 (0.8)	207

Percentages are based on the number of non-missing questionnaires. The questionnaire should have been completed prior to infusion on day 1 of each cycle and at the off-treatment visit. The questionnaire was not filled out on first day of Cycle 1 if completed at baseline.

Table S6. EQ-VAS health state scores**.**

Cycle		Absolute value	Change from baseline
Baseline[a]	n	279	NA
	Mean (SD)	60.5 (19.0)	NA
	Median	60.0	NA
	Range	7–100	NA
1	n	72	58
	Mean (SD)	63 (19.2)	0.9 (10.1)
	Median	61.0	0.0
	Range	10–100	-20–30
2	n	224	202
	Mean (SD)	61.5 (18.7)	-0.2 (15.7)
	Median	60	0.0
	Range	10–100	-84–50
3	n	189	171
	Mean (SD)	62.5 (18.6)	0.4 (15.9)
	Median	62.0	0.0
	Range	20–100	-60–50
4	n	157	140
	Mean (SD)	62.8 (18.1)	-0.1 (16.7)
	Median	60.0	0.0
	Range	20–99	-58–40
5	n	127	114
	Mean (SD)	63.3 (18.1)	-1.5 (16.1)
	Median	65	0.0
	Range	15–99	-58–50

The questionnaire should have been completed prior to infusion on Day 1 of each cycle and at the off-treatment visit. The questionnaire was not filled out on first day of Cycle 1 if completed at baseline. Health state scores range from 0 to 100, where 0 = worst health imaginable and 100 = best health imaginable.

[a]Baseline data were unavailable for 14 patients.

BL, baseline; NA, not applicable; SAS, safety analysis set; SD, standard deviation; VAS, visual analogue scale.

Table S7. Patient Neurotoxic Questionnaire (patients with EIPN).

Cycle	Patients with at least 1 EIPN (N = 108)					
	**A[a] no**	**B[a] mild**	**C[a] moderate**	**D[a] moderate to severe**	**E[a] severe**	**Missing[b]**
	Item 1: Numbness, pain or tingling in hands and feet, n (%)					
Baseline	39 (40.6)	33 (34.4)	12 (12.5)	10 (10.4)	2 (2.1)	12 (11.1)
1	7 (26.9)	11 (42.3)	3 (11.5)	4 (15.4)	1 (3.8)	82 (75.9)
2	32 (37.2)	30 (34.9)	10 (11.6)	13 (15.1)	1 (1.2)	22 (20.4)
3	21 (26.6)	37 (46.8)	10 (12.7)	9 (11.4)	2 (2.5)	29 (26.9)
4	13 (19.1)	23 (33.8)	17 (25.0)	13 (19.1)	2 (2.9)	40 (37.0)
5	12 (20.0)	25 (41.7)	14 (23.3)	9 (15.0)	0	48 (44.4)
	Item 2: Weakness in arms or legs, n (%)					
Baseline	36 (37.1%)	24 (24.7%)	16 (16.5%)	19 (19.6%)	2 (2.1%)	11 (10.2)
1	5 (20.0%)	8 (32.0%)	6 (24.0%)	4 (16.0%)	2 (8.0%)	83 (76.9)
2	23 (27.1%)	34 (40.0%)	11 (12.9%)	16 (18.8%)	1 (1.2%)	23 (21.3)
3	22 (27.5%)	30 (37.5%)	9 (11.3%)	17 (21.3%)	2 (2.5%)	28 (25.9)
4	13 (19.1%)	20 (29.4%)	13 (19.1%)	20 (29.4%)	2 (2.9%)	40 (37.0)
5	14 (24.1%)	19 (32.8%)	12 (20.7%)	12 (20.7%)	1 (1.7%)	50 (46.3)

The questionnaire was not filled out on first day of Cycle 1 if completed at baseline.

[a]Percentages are based on the number of nonmissing questionnaires; [b]percentages are based on the total population. EIPN, eribulin-induced peripheral neuropathy.

Figure S1. Patient disposition.

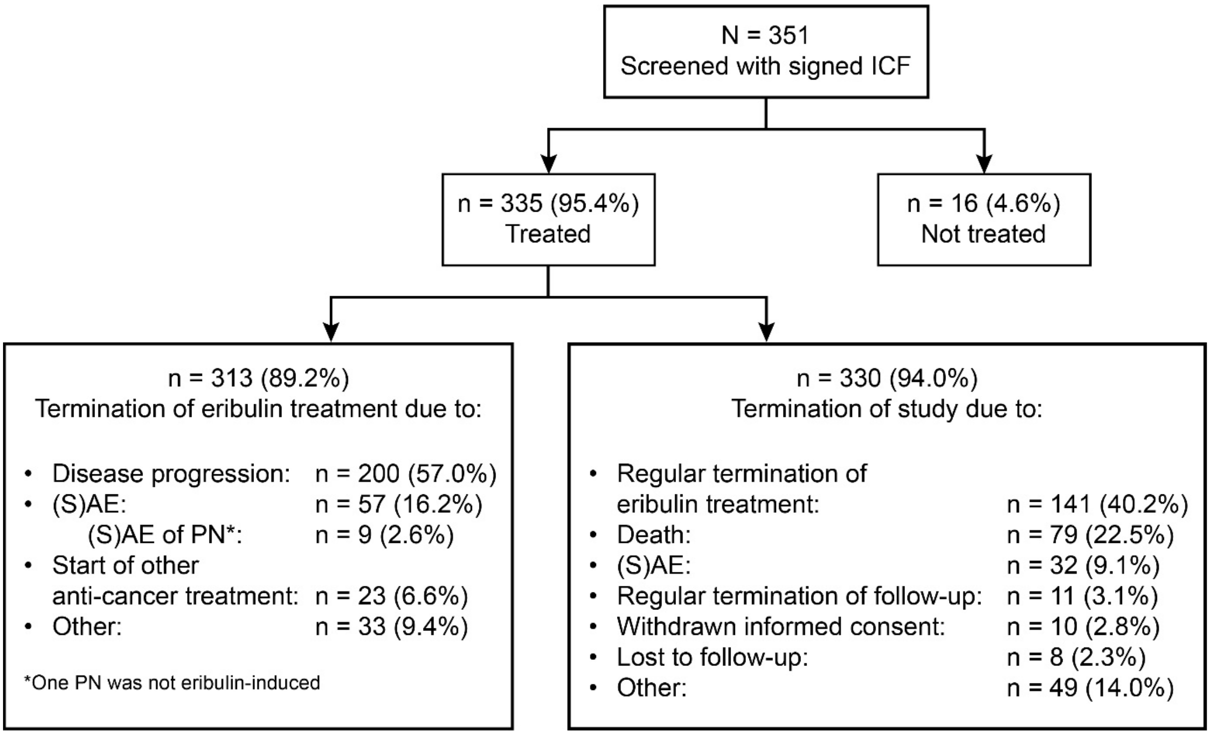

Percentages are based on the screened population (N = 351). Patient disposition for this observational study reflects the collected case report form documentation. At data cutoff, an end of study case report form was unavailable for 5 treated patients; thus, no termination of study reason is available for these patients.

(S)AE, (serious) adverse event; ICF, informed consent form; PN, peripheral neuropathy.

Figure S2. Kaplan–Meier plot for time to improvement of EIPN by presence (red line) or absence (blue line) of therapeutic intervention.

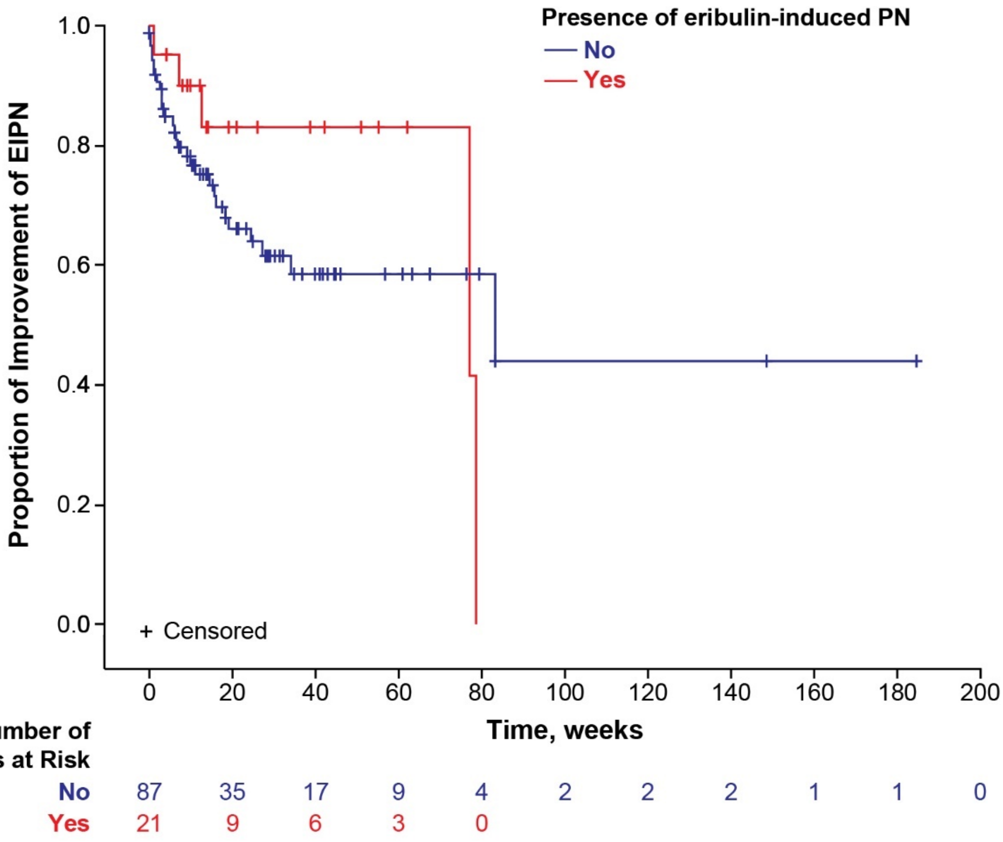

EIPN, eribulin-induced peripheral neuropathy.

Figure S3. Kaplan–Meier plot for time to disease progression by presence (red line) or absence (blue line) of EIPN.

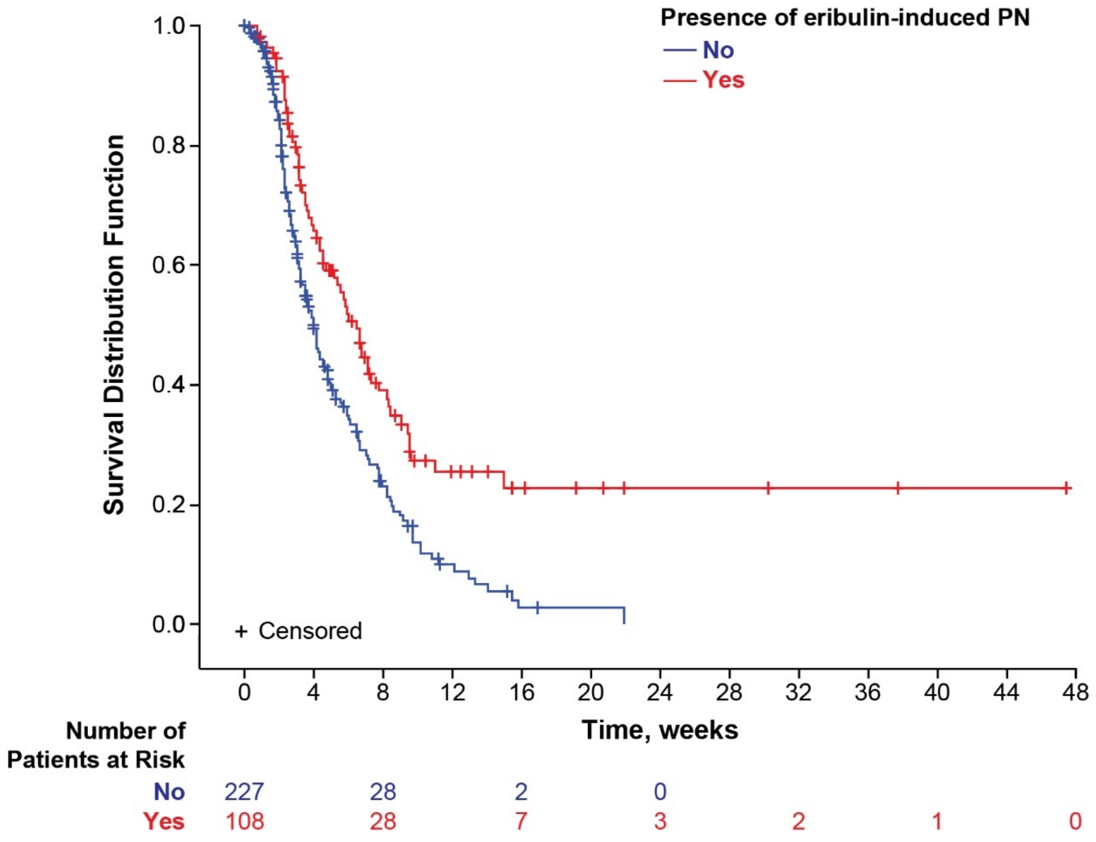

EIPN, eribulin-induced peripheral neuropathy.
